# Supplementary material for: Modeling glioblastoma heterogeneity as a dynamic network of cell states
Source: Mol Syst Biol. 2021 Sep 16;17(9):e10105. doi: 10.15252/msb.202010105 (PMC8444284; doi:10.15252/msb.202010105)
Supplement: Supplementary file 6 — Source Data for Figure 5 [file MSB-17-e10105-s004.zip › Figure5A_sourcedata/GSEA_3071/nosigdb_stateIII.GseaPreranked.1622795155562/gsea_report_for_na_neg_1622795155562.html]

Report for na\_neg 1622795155562 [GSEA]

| GS  follow link to MSigDB | GS DETAILS | SIZE | ES | NES | NOM p-val | FDR q-val | FWER p-val | RANK AT MAX | LEADING EDGE || 1 | NB\_KI\_CLUSTER18 | Details ... | 53 | -0.64 | -3.47 | 0.000 | 0.000 | 0.000 | 357 | tags=51%, list=9%, signal=55% |
| 2 | OLSEN\_SCPLIKE | Details ... | 50 | -0.64 | -3.41 | 0.000 | 0.000 | 0.000 | 361 | tags=54%, list=9%, signal=59% |
| 3 | ANASTASSIOU\_MULTICANCER\_INVASIVENESS\_PMID\_22208948 | Details ... | 39 | -0.68 | -3.40 | 0.000 | 0.000 | 0.000 | 257 | tags=56%, list=7%, signal=60% |
| 4 | FELDMAN\_2020\_M-EARLYG1 | Details ... | 57 | -0.61 | -3.37 | 0.000 | 0.000 | 0.000 | 585 | tags=56%, list=15%, signal=65% |
| 5 | OLSEN\_MESENCHYMAL | Details ... | 60 | -0.60 | -3.35 | 0.000 | 0.000 | 0.000 | 340 | tags=50%, list=9%, signal=54% |
| 6 | LARSSON\_STATE\_3 | Details ... | 49 | -0.62 | -3.33 | 0.000 | 0.000 | 0.000 | 408 | tags=55%, list=10%, signal=61% |
| 7 | IVY\_CTMVP | Details ... | 60 | -0.59 | -3.24 | 0.000 | 0.000 | 0.000 | 580 | tags=55%, list=15%, signal=64% |
| 8 | RICHARDS\_ZHONG\_NATURE\_2018\_OPC\_UPREG | Details ... | 42 | -0.63 | -3.24 | 0.000 | 0.000 | 0.000 | 445 | tags=57%, list=11%, signal=64% |
| 9 | DOROSZKO\_U3013MG\_CLUSTER\_0 | Details ... | 19 | -0.78 | -3.15 | 0.000 | 0.000 | 0.000 | 359 | tags=74%, list=9%, signal=81% |
| 10 | DOROSZKO\_U3054MG\_CLUSTER\_2 | Details ... | 176 | -0.47 | -3.14 | 0.000 | 0.000 | 0.000 | 580 | tags=41%, list=15%, signal=46% |
| 11 | LARSSON\_STATE\_2 | Details ... | 52 | -0.59 | -3.13 | 0.000 | 0.000 | 0.000 | 415 | tags=50%, list=11%, signal=55% |
| 12 | IVY\_CT | Details ... | 96 | -0.50 | -3.11 | 0.000 | 0.000 | 0.000 | 579 | tags=46%, list=15%, signal=52% |
| 13 | DOROSZKO\_PERIVASCULAR\_INVASION | Details ... | 25 | -0.70 | -3.04 | 0.000 | 0.000 | 0.000 | 375 | tags=60%, list=10%, signal=66% |
| 14 | RICHARDS\_ZHONG\_NATURE\_2018\_ASTROCYTES\_UPREG | Details ... | 135 | -0.46 | -2.97 | 0.000 | 0.000 | 0.000 | 444 | tags=38%, list=11%, signal=41% |
| 15 | RICHARDS\_NOWAKOWSKI\_SCIENCE\_2017\_OPC\_UPREG | Details ... | 170 | -0.44 | -2.89 | 0.000 | 0.000 | 0.000 | 465 | tags=37%, list=12%, signal=40% |
| 16 | DOROSZKO\_U3054MG\_CLUSTER\_0 | Details ... | 45 | -0.55 | -2.88 | 0.000 | 0.000 | 0.000 | 1119 | tags=71%, list=28%, signal=98% |
| 17 | RICHARDS\_NOWAKOWSKI\_SCIENCE\_2017\_ASTROCYTE\_UPREG | Details ... | 264 | -0.41 | -2.86 | 0.000 | 0.000 | 0.000 | 509 | tags=34%, list=13%, signal=37% |
| 18 | SUVA\_AC\_PMID\_31327527 | Details ... | 18 | -0.72 | -2.83 | 0.000 | 0.000 | 0.000 | 415 | tags=67%, list=11%, signal=74% |
| 19 | ALMSTEDT\_VGR\_DIFF | Details ... | 311 | -0.39 | -2.82 | 0.000 | 0.000 | 0.000 | 444 | tags=32%, list=11%, signal=33% |
| 20 | FELDMAN\_2020\_G1 | Details ... | 59 | -0.50 | -2.81 | 0.000 | 0.000 | 0.000 | 306 | tags=34%, list=8%, signal=36% |
| 21 | TISIG\_PMID\_32649888 |  | 28 | -0.62 | -2.80 | 0.000 | 0.000 | 0.000 | 498 | tags=61%, list=13%, signal=69% |
| 22 | RICHARDS\_NEFTEL\_CELL\_2019\_AC |  | 18 | -0.72 | -2.80 | 0.000 | 0.000 | 0.000 | 415 | tags=67%, list=11%, signal=74% |
| 23 | FELDMAN\_2020\_LATEG1 |  | 44 | -0.54 | -2.79 | 0.000 | 0.000 | 0.000 | 568 | tags=50%, list=14%, signal=58% |
| 24 | GANDAL\_IBD\_UP\_PMID29439242 |  | 44 | -0.55 | -2.78 | 0.000 | 0.000 | 0.000 | 588 | tags=50%, list=15%, signal=58% |
| 25 | FELDMAN\_2020\_NEURAL G0 |  | 177 | -0.40 | -2.70 | 0.000 | 0.000 | 0.000 | 388 | tags=29%, list=10%, signal=31% |
| 26 | ALLEN\_113\_ASTROL1FGFR3FOS |  | 18 | -0.70 | -2.70 | 0.000 | 0.000 | 0.000 | 566 | tags=67%, list=14%, signal=78% |
| 27 | TIISIG\_PMID\_32649888 |  | 17 | -0.71 | -2.68 | 0.000 | 0.000 | 0.000 | 314 | tags=59%, list=8%, signal=64% |
| 28 | PARADA\_T1\_UP\_26461091 |  | 30 | -0.57 | -2.67 | 0.000 | 0.000 | 0.000 | 734 | tags=60%, list=19%, signal=73% |
| 29 | ALLEN\_112\_ASTROL1FGFR3MT1G |  | 20 | -0.65 | -2.63 | 0.000 | 0.000 | 0.000 | 744 | tags=70%, list=19%, signal=86% |
| 30 | VANGRONINGEN\_MES |  | 186 | -0.39 | -2.62 | 0.000 | 0.000 | 0.000 | 498 | tags=33%, list=13%, signal=36% |
| 31 | ALLEN\_111\_ASTROL1\_6FGFR3ETNPPL |  | 18 | -0.66 | -2.58 | 0.000 | 0.000 | 0.000 | 566 | tags=67%, list=14%, signal=78% |
| 32 | RICHARDS\_NOWAKOWSKI\_SCIENCE\_2017\_NIN1\_UPREG |  | 81 | -0.43 | -2.56 | 0.000 | 0.000 | 0.000 | 830 | tags=48%, list=21%, signal=60% |
| 33 | RICHARDS\_NOWAKOWSKI\_SCIENCE\_2017\_VRG\_UPREG |  | 187 | -0.38 | -2.54 | 0.000 | 0.000 | 0.000 | 449 | tags=31%, list=11%, signal=33% |
| 34 | RICHARDS\_VERHAAK\_CANCERCELL\_2010\_PRONEURAL |  | 60 | -0.45 | -2.53 | 0.000 | 0.000 | 0.000 | 359 | tags=35%, list=9%, signal=38% |
| 35 | GANDAL\_BIPOLAR\_UP\_PMID29439242 |  | 33 | -0.54 | -2.53 | 0.000 | 0.000 | 0.000 | 415 | tags=48%, list=11%, signal=54% |
| 36 | KLING\_GBM\_PHENO\_PMID\_27667176 |  | 77 | -0.43 | -2.48 | 0.000 | 0.000 | 0.000 | 498 | tags=35%, list=13%, signal=39% |
| 37 | RICHARDS\_NOWAKOWSKI\_SCIENCE\_2017\_NIN3\_UPREG |  | 71 | -0.42 | -2.47 | 0.000 | 0.000 | 0.000 | 773 | tags=46%, list=20%, signal=57% |
| 38 | VANGRONINGEN\_ADRN |  | 125 | -0.38 | -2.46 | 0.000 | 0.000 | 0.000 | 438 | tags=32%, list=11%, signal=35% |
| 39 | SINGH\_SOX2\_UPREGULATED\_TRANSCRIPTS\_PMID\_28122245 |  | 184 | -0.36 | -2.44 | 0.000 | 0.000 | 0.000 | 1162 | tags=50%, list=30%, signal=68% |
| 40 | OUTERRADIALGLIA |  | 32 | -0.51 | -2.43 | 0.000 | 0.000 | 0.000 | 421 | tags=41%, list=11%, signal=45% |
| 41 | POLLEN\_OUTERRADIALGLIA\_PMID\_26406371 |  | 32 | -0.51 | -2.42 | 0.000 | 0.000 | 0.000 | 421 | tags=41%, list=11%, signal=45% |
| 42 | GAROFANO\_IAVARONE\_LIST\_OF\_NEURAL\_PROGENITOR\_MARKER\_GENES |  | 36 | -0.50 | -2.41 | 0.000 | 0.000 | 0.001 | 371 | tags=39%, list=9%, signal=43% |
| 43 | DOROSZKO\_U3054MG\_CLUSTER\_5 |  | 56 | -0.44 | -2.41 | 0.000 | 0.000 | 0.001 | 940 | tags=55%, list=24%, signal=72% |
| 44 | SUVA\_NPC1\_PMID\_31327527 |  | 24 | -0.54 | -2.38 | 0.000 | 0.000 | 0.005 | 568 | tags=50%, list=14%, signal=58% |
| 45 | IVY\_IT |  | 30 | -0.51 | -2.37 | 0.000 | 0.000 | 0.005 | 775 | tags=53%, list=20%, signal=66% |
| 46 | DOROSZKO\_U3180MG\_CLUSTER\_2 |  | 153 | -0.35 | -2.35 | 0.000 | 0.000 | 0.007 | 905 | tags=40%, list=23%, signal=50% |
| 47 | RICHARDS\_NOWAKOWSKI\_SCIENCE\_2017\_EN\_PFC3\_UPREG |  | 108 | -0.37 | -2.35 | 0.000 | 0.000 | 0.007 | 1020 | tags=48%, list=26%, signal=63% |
| 48 | RICHARDS\_NEFTEL\_CELL\_2019\_NPC1 |  | 24 | -0.54 | -2.28 | 0.000 | 0.000 | 0.012 | 568 | tags=50%, list=14%, signal=58% |
| 49 | FELDMAN\_2020\_G2-M |  | 114 | -0.36 | -2.28 | 0.000 | 0.000 | 0.014 | 617 | tags=32%, list=16%, signal=37% |
| 50 | KRONA\_ROSEN\_WHITE\_MATTER\_INVASION |  | 126 | -0.35 | -2.26 | 0.000 | 0.000 | 0.017 | 706 | tags=36%, list=18%, signal=42% |
| 51 | RICHARDS\_VERHAAK\_CANCERCELL\_2010\_CLASSICAL |  | 64 | -0.40 | -2.24 | 0.000 | 0.000 | 0.020 | 923 | tags=50%, list=23%, signal=64% |
| 52 | NB\_KI\_CLUSTER17 |  | 68 | -0.39 | -2.23 | 0.000 | 0.000 | 0.028 | 1019 | tags=51%, list=26%, signal=68% |
| 53 | DALMO\_BARCODENUMBER\_UNPUB |  | 88 | -0.37 | -2.23 | 0.000 | 0.000 | 0.028 | 719 | tags=39%, list=18%, signal=46% |
| 54 | RICHARDS\_NEFTEL\_CELL\_2019\_OPC |  | 19 | -0.56 | -2.22 | 0.000 | 0.000 | 0.030 | 190 | tags=37%, list=5%, signal=39% |
| 55 | GANDAL\_SCHIZOPHRENIA\_UP\_PMID29439242 |  | 46 | -0.43 | -2.22 | 0.000 | 0.000 | 0.030 | 1052 | tags=54%, list=27%, signal=73% |
| 56 | WANG\_MESENCHYMAL\_PMID\_28697342 |  | 22 | -0.53 | -2.21 | 0.000 | 0.000 | 0.031 | 614 | tags=50%, list=16%, signal=59% |
| 57 | SUVA\_OPC\_PMID\_31327527 |  | 19 | -0.56 | -2.21 | 0.000 | 0.000 | 0.031 | 190 | tags=37%, list=5%, signal=39% |
| 58 | RICHARDS\_NOWAKOWSKI\_SCIENCE\_2017\_IN\_CTX\_CGE2\_UPREG |  | 98 | -0.35 | -2.20 | 0.000 | 0.000 | 0.033 | 509 | tags=32%, list=13%, signal=35% |
| 59 | DALMO\_EXTINCTION\_UNPUB |  | 22 | -0.53 | -2.18 | 0.000 | 0.001 | 0.038 | 614 | tags=50%, list=16%, signal=59% |
| 60 | RICHARDS\_NOWAKOWSKI\_SCIENCE\_2017\_MGE\_IPC3\_UPREG |  | 63 | -0.39 | -2.18 | 0.000 | 0.001 | 0.039 | 382 | tags=32%, list=10%, signal=35% |
| 61 | ALMSTEDT\_ALK |  | 35 | -0.43 | -2.14 | 0.000 | 0.001 | 0.047 | 970 | tags=49%, list=25%, signal=64% |
| 62 | FELDMAN\_2020\_S-G2 |  | 125 | -0.33 | -2.13 | 0.000 | 0.001 | 0.050 | 418 | tags=24%, list=11%, signal=26% |
| 63 | RICHARDS\_NOWAKOWSKI\_SCIENCE\_2017\_IN\_STR\_UPREG |  | 56 | -0.39 | -2.12 | 0.001 | 0.001 | 0.053 | 613 | tags=38%, list=16%, signal=44% |
| 64 | RICHARDS\_NEFTEL\_CELL\_2019\_NPC2 |  | 17 | -0.55 | -2.11 | 0.000 | 0.001 | 0.059 | 433 | tags=47%, list=11%, signal=53% |
| 65 | RICHARDS\_NOWAKOWSKI\_SCIENCE\_2017\_MGE\_RG1\_UPREG |  | 144 | -0.32 | -2.09 | 0.000 | 0.001 | 0.069 | 510 | tags=28%, list=13%, signal=32% |
| 66 | ALLEN\_115\_OLIGOL4\_6MOBPCOL18A1 |  | 17 | -0.55 | -2.09 | 0.002 | 0.001 | 0.070 | 307 | tags=41%, list=8%, signal=44% |
| 67 | DOROSZKO\_U3013MG\_CLUSTER\_3 |  | 36 | -0.44 | -2.09 | 0.000 | 0.001 | 0.072 | 389 | tags=33%, list=10%, signal=37% |
| 68 | RICHARDS\_NEFTEL\_CELL\_2019\_MES1 |  | 21 | -0.51 | -2.08 | 0.002 | 0.001 | 0.075 | 567 | tags=43%, list=14%, signal=50% |
| 69 | SUVA\_MES1\_PMID\_31327527 |  | 21 | -0.51 | -2.08 | 0.000 | 0.001 | 0.078 | 567 | tags=43%, list=14%, signal=50% |
| 70 | RICHARDS\_NOWAKOWSKI\_SCIENCE\_2017\_EN\_PFC1\_UPREG |  | 322 | -0.29 | -2.08 | 0.000 | 0.001 | 0.082 | 940 | tags=38%, list=24%, signal=45% |
| 71 | SUVA\_NPC2\_PMID\_31327527 |  | 17 | -0.55 | -2.07 | 0.000 | 0.001 | 0.094 | 433 | tags=47%, list=11%, signal=53% |
| 72 | RICHARDS\_NOWAKOWSKI\_SCIENCE\_2017\_IPC\_NEN1\_UPREG |  | 114 | -0.33 | -2.05 | 0.000 | 0.001 | 0.107 | 1355 | tags=54%, list=34%, signal=79% |
| 73 | DOROSZKO\_U3054MG\_CLUSTER\_10 |  | 99 | -0.32 | -2.00 | 0.001 | 0.002 | 0.167 | 834 | tags=35%, list=21%, signal=44% |
| 74 | RICHARDS\_NOWAKOWSKI\_SCIENCE\_2017\_NEN\_EARLY2\_UPREG |  | 172 | -0.30 | -2.00 | 0.000 | 0.002 | 0.169 | 1270 | tags=49%, list=32%, signal=69% |
| 75 | IVY\_CTPAN |  | 68 | -0.35 | -2.00 | 0.001 | 0.002 | 0.171 | 310 | tags=22%, list=8%, signal=24% |
| 76 | IVY\_LE |  | 27 | -0.44 | -1.96 | 0.005 | 0.003 | 0.221 | 731 | tags=48%, list=19%, signal=59% |
| 77 | GANDAL\_ALCOHOLADDICTION\_DN\_PMID29439242 |  | 42 | -0.39 | -1.96 | 0.003 | 0.003 | 0.221 | 760 | tags=40%, list=19%, signal=50% |
| 78 | GANDAL\_AUTISM\_UP\_PMID29439242 |  | 48 | -0.37 | -1.95 | 0.001 | 0.003 | 0.235 | 170 | tags=21%, list=4%, signal=22% |
| 79 | DOROSZKO\_U3013MG\_CLUSTER\_1 |  | 16 | -0.51 | -1.95 | 0.003 | 0.003 | 0.235 | 188 | tags=31%, list=5%, signal=33% |
| 80 | LARSSON\_STATE\_6 |  | 45 | -0.38 | -1.94 | 0.003 | 0.004 | 0.266 | 371 | tags=27%, list=9%, signal=29% |
| 81 | RICHARDS\_CAHOY\_JNEUROSCI\_2016\_CULTURED\_ASTROGLIA |  | 436 | -0.26 | -1.92 | 0.000 | 0.004 | 0.320 | 479 | tags=22%, list=12%, signal=22% |
| 82 | RICHARDS\_NOWAKOWSKI\_SCIENCE\_2017\_IN\_CTX\_MGE1\_UPREG |  | 116 | -0.30 | -1.91 | 0.002 | 0.004 | 0.322 | 892 | tags=38%, list=23%, signal=48% |
| 83 | RICHARDS\_NOWAKOWSKI\_SCIENCE\_2017\_U1\_UPREG |  | 451 | -0.26 | -1.91 | 0.000 | 0.005 | 0.348 | 450 | tags=22%, list=11%, signal=22% |
| 84 | DOROSZKO\_U3180MG\_CLUSTER\_5 |  | 22 | -0.45 | -1.89 | 0.005 | 0.005 | 0.395 | 206 | tags=27%, list=5%, signal=29% |
| 85 | DOROSZKO\_U3054MG\_CLUSTER\_7 |  | 20 | -0.46 | -1.88 | 0.014 | 0.006 | 0.406 | 1145 | tags=65%, list=29%, signal=91% |
| 86 | RICHARDS\_CAHOY\_JNEUROSCI\_2010\_ASTRO\_YOUNG |  | 297 | -0.26 | -1.83 | 0.001 | 0.009 | 0.558 | 458 | tags=23%, list=12%, signal=24% |
| 87 | PARADA\_T2\_UP\_26461091 |  | 34 | -0.38 | -1.82 | 0.008 | 0.009 | 0.585 | 1026 | tags=47%, list=26%, signal=63% |
| 88 | RICHARDS\_LIDDELOW\_NATURE\_2017\_A2\_REACTIVE\_ASTROCYTES |  | 18 | -0.47 | -1.82 | 0.008 | 0.010 | 0.593 | 191 | tags=28%, list=5%, signal=29% |
| 89 | GAROFANO\_IAVARONE\_NEU\_SUBTYPE |  | 105 | -0.29 | -1.81 | 0.006 | 0.010 | 0.613 | 851 | tags=35%, list=22%, signal=44% |
| 90 | RICHARDS\_NOWAKOWSKI\_SCIENCE\_2017\_ORG\_UPREG |  | 475 | -0.24 | -1.80 | 0.000 | 0.010 | 0.628 | 480 | tags=21%, list=12%, signal=21% |
| 91 | RICHARDS\_NOWAKOWSKI\_SCIENCE\_2017\_MGE\_IPC2\_UPREG |  | 193 | -0.26 | -1.79 | 0.001 | 0.011 | 0.662 | 894 | tags=34%, list=23%, signal=42% |
| 92 | ALLEN\_116\_OLIGOL4\_6OPALIN |  | 16 | -0.47 | -1.78 | 0.013 | 0.012 | 0.693 | 865 | tags=50%, list=22%, signal=64% |
| 93 | WENG\_STEMNESS\_PMID\_30982771 |  | 22 | -0.44 | -1.78 | 0.010 | 0.012 | 0.700 | 894 | tags=50%, list=23%, signal=64% |
| 94 | RICHARDS\_NOWAKOWSKI\_SCIENCE\_2017\_IN\_CTX\_MGE2\_UPREG |  | 97 | -0.29 | -1.78 | 0.005 | 0.012 | 0.702 | 1019 | tags=40%, list=26%, signal=53% |
| 95 | RICHARDS\_NOWAKOWSKI\_SCIENCE\_2017\_MURAL\_UPREG |  | 307 | -0.25 | -1.77 | 0.000 | 0.013 | 0.720 | 467 | tags=22%, list=12%, signal=23% |
| 96 | RICHARDS\_VERHAAK\_CANCERCELL\_2010\_MESENCHYMAL |  | 54 | -0.32 | -1.77 | 0.012 | 0.013 | 0.737 | 1750 | tags=67%, list=45%, signal=119% |
| 97 | RICHARDS\_NOWAKOWSKI\_SCIENCE\_2017\_TRG\_UPREG |  | 385 | -0.24 | -1.75 | 0.000 | 0.015 | 0.788 | 371 | tags=18%, list=9%, signal=18% |
| 98 | DOROSZKO\_U3054MG\_CLUSTER\_3 |  | 83 | -0.29 | -1.73 | 0.014 | 0.017 | 0.822 | 1253 | tags=47%, list=32%, signal=68% |
| 99 | RICHARDS\_NOWAKOWSKI\_SCIENCE\_2017\_IN\_CTX\_CGE1\_UPREG |  | 92 | -0.28 | -1.73 | 0.006 | 0.017 | 0.827 | 817 | tags=34%, list=21%, signal=42% |
| 100 | RICHARDS\_NOWAKOWSKI\_SCIENCE\_2017\_EN\_PFC2\_UPREG |  | 336 | -0.24 | -1.73 | 0.000 | 0.017 | 0.830 | 828 | tags=31%, list=21%, signal=36% |
| 101 | KRONA\_ROSEN\_PERIVASCULAR\_INVASION |  | 31 | -0.36 | -1.67 | 0.027 | 0.025 | 0.928 | 1014 | tags=48%, list=26%, signal=65% |
| 102 | SINGH\_SOX2\_DOWNREGULATED\_TRANSCRIPTS\_PMID\_28122245 |  | 63 | -0.30 | -1.66 | 0.026 | 0.026 | 0.938 | 537 | tags=29%, list=14%, signal=33% |
| 103 | RICHARDS\_NOWAKOWSKI\_SCIENCE\_2017\_NIN5\_UPREG |  | 99 | -0.26 | -1.66 | 0.016 | 0.026 | 0.941 | 1033 | tags=38%, list=26%, signal=51% |
| 104 | NB\_KI\_CLUSTER4 |  | 42 | -0.33 | -1.65 | 0.029 | 0.028 | 0.946 | 511 | tags=31%, list=13%, signal=35% |
| 105 | ROSEN\_KRONA\_TI\_UNPUBL |  | 27 | -0.37 | -1.64 | 0.039 | 0.029 | 0.958 | 1272 | tags=59%, list=32%, signal=87% |
| 106 | RICHARDS\_CAHOY\_JNEUROSCI\_2014\_MOG\_POS |  | 256 | -0.23 | -1.60 | 0.004 | 0.038 | 0.986 | 1206 | tags=39%, list=31%, signal=52% |
| 107 | RICHARDS\_NOWAKOWSKI\_SCIENCE\_2017\_NIN4\_UPREG |  | 58 | -0.29 | -1.60 | 0.025 | 0.038 | 0.988 | 378 | tags=22%, list=10%, signal=24% |
| 108 | NB\_KI\_CLUSTER23 |  | 82 | -0.27 | -1.59 | 0.027 | 0.041 | 0.992 | 484 | tags=26%, list=12%, signal=29% |
| 109 | RICHARDS\_NOWAKOWSKI\_SCIENCE\_2017\_RG\_DIV2\_UPREG |  | 446 | -0.22 | -1.58 | 0.003 | 0.042 | 0.992 | 905 | tags=30%, list=23%, signal=34% |
| 110 | RICHARDS\_CAHOY\_JNEUROSCI\_2008\_OLIGODENDROCYTE |  | 382 | -0.22 | -1.57 | 0.001 | 0.045 | 0.994 | 990 | tags=32%, list=25%, signal=39% |
| 111 | RICHARDS\_ZHONG\_NATURE\_2018\_MICROGLIA\_UPREG |  | 105 | -0.25 | -1.57 | 0.034 | 0.045 | 0.994 | 1217 | tags=42%, list=31%, signal=59% |
| 112 | OLSEN\_ADRENERGIC |  | 41 | -0.31 | -1.56 | 0.050 | 0.048 | 0.999 | 731 | tags=34%, list=19%, signal=42% |
| 113 | RICHARDS\_CAHOY\_JNEUROSCI\_2009\_NEURON |  | 337 | -0.21 | -1.52 | 0.002 | 0.061 | 1.000 | 848 | tags=29%, list=22%, signal=34% |
| 114 | RICHARDS\_NOWAKOWSKI\_SCIENCE\_2017\_IPC\_NEN1\_UPREG\_1 |  | 71 | -0.26 | -1.50 | 0.058 | 0.068 | 1.000 | 332 | tags=20%, list=8%, signal=21% |
| 115 | SEURAT\_CELLCYCLE |  | 39 | -0.29 | -1.48 | 0.081 | 0.075 | 1.000 | 1242 | tags=49%, list=32%, signal=71% |
| 116 | RICHARDS\_CAHOY\_JNEUROSCI\_2013\_OL\_MYEL |  | 380 | -0.20 | -1.46 | 0.008 | 0.085 | 1.000 | 1145 | tags=36%, list=29%, signal=46% |
| 117 | NB\_KI\_CLUSTER16 |  | 45 | -0.28 | -1.45 | 0.081 | 0.088 | 1.000 | 1246 | tags=47%, list=32%, signal=68% |
| 118 | LARSSON\_STATE\_4 |  | 34 | -0.30 | -1.44 | 0.095 | 0.095 | 1.000 | 463 | tags=24%, list=12%, signal=26% |
| 119 | DOROSZKO\_U3054MG\_CLUSTER\_6 |  | 42 | -0.28 | -1.40 | 0.117 | 0.114 | 1.000 | 1370 | tags=50%, list=35%, signal=76% |
| 120 | RICHARDS\_NOWAKOWSKI\_SCIENCE\_2017\_IPC\_DIV1\_UPREG |  | 232 | -0.20 | -1.39 | 0.054 | 0.119 | 1.000 | 1072 | tags=33%, list=27%, signal=42% |
| 121 | RICHARDS\_NOWAKOWSKI\_SCIENCE\_2017\_NEN\_LATE\_UPREG |  | 116 | -0.22 | -1.39 | 0.089 | 0.121 | 1.000 | 891 | tags=33%, list=23%, signal=41% |
| 122 | NB\_KI\_CLUSTER6 |  | 90 | -0.23 | -1.39 | 0.099 | 0.122 | 1.000 | 1328 | tags=47%, list=34%, signal=69% |
| 123 | NB\_KI\_CLUSTER14 |  | 96 | -0.23 | -1.37 | 0.121 | 0.132 | 1.000 | 2108 | tags=67%, list=54%, signal=140% |
| 124 | RICHARDS\_NOWAKOWSKI\_SCIENCE\_2017\_NEN\_EARLY1\_UPREG |  | 226 | -0.19 | -1.35 | 0.069 | 0.145 | 1.000 | 957 | tags=31%, list=24%, signal=38% |
| 125 | SEGERMAN\_MULTITHERAPY\_RESISTANCE\_FIG5A\_PMID\_27974212 |  | 15 | -0.37 | -1.35 | 0.137 | 0.145 | 1.000 | 1976 | tags=80%, list=50%, signal=160% |
| 126 | RICHARDS\_ZHONG\_NATURE\_2018\_NPCS\_UPREG |  | 50 | -0.25 | -1.34 | 0.150 | 0.149 | 1.000 | 463 | tags=20%, list=12%, signal=22% |
| 127 | RICHARDS\_NOWAKOWSKI\_SCIENCE\_2017\_EN\_V1\_2\_UPREG |  | 281 | -0.19 | -1.32 | 0.060 | 0.163 | 1.000 | 828 | tags=27%, list=21%, signal=32% |
| 128 | NB\_KI\_CLUSTER8 |  | 79 | -0.22 | -1.32 | 0.140 | 0.167 | 1.000 | 1301 | tags=44%, list=33%, signal=65% |
| 129 | RICHARDS\_NOWAKOWSKI\_SCIENCE\_2017\_EN\_V1\_3\_UPREG |  | 317 | -0.18 | -1.29 | 0.082 | 0.191 | 1.000 | 453 | tags=18%, list=12%, signal=19% |
| 130 | GANDAL\_ALCOHOLADDICTION\_UP\_PMID29439242 |  | 48 | -0.24 | -1.29 | 0.201 | 0.191 | 1.000 | 1000 | tags=38%, list=25%, signal=50% |
| 131 | RICHARDS\_CAHOY\_JNEUROSCI\_2011\_ASTRO\_MATURE |  | 215 | -0.18 | -1.26 | 0.142 | 0.218 | 1.000 | 775 | tags=24%, list=20%, signal=28% |
| 132 | ALMSTEDT\_11Q\_MSIGDB |  | 119 | -0.19 | -1.23 | 0.207 | 0.242 | 1.000 | 1045 | tags=32%, list=27%, signal=42% |
| 133 | PARADA\_T1\_DN\_26461091 |  | 27 | -0.27 | -1.22 | 0.264 | 0.258 | 1.000 | 752 | tags=33%, list=19%, signal=41% |
| 134 | DOROSZKO\_U3180MG\_CLUSTER\_4 |  | 42 | -0.23 | -1.19 | 0.254 | 0.283 | 1.000 | 1370 | tags=45%, list=35%, signal=69% |
| 135 | RICHARDS\_NOWAKOWSKI\_SCIENCE\_2017\_EN\_V1\_1\_UPREG |  | 336 | -0.17 | -1.18 | 0.171 | 0.296 | 1.000 | 941 | tags=28%, list=24%, signal=34% |
| 136 | RICHARDS\_NOWAKOWSKI\_SCIENCE\_2017\_MICROGLIA\_UPREG |  | 270 | -0.17 | -1.17 | 0.227 | 0.301 | 1.000 | 1015 | tags=30%, list=26%, signal=38% |
| 137 | DOROSZKO\_U3180MG\_CLUSTER\_6 |  | 16 | -0.31 | -1.17 | 0.286 | 0.300 | 1.000 | 1671 | tags=63%, list=43%, signal=108% |
| 138 | NB\_KI\_CLUSTER15 |  | 106 | -0.18 | -1.16 | 0.301 | 0.320 | 1.000 | 1818 | tags=55%, list=46%, signal=99% |
| 139 | NB\_KI\_CLUSTER2 |  | 91 | -0.19 | -1.15 | 0.290 | 0.320 | 1.000 | 2362 | tags=70%, list=60%, signal=172% |
| 140 | DOROSZKO\_U3013MG\_CLUSTER\_2 |  | 63 | -0.20 | -1.14 | 0.313 | 0.329 | 1.000 | 1371 | tags=41%, list=35%, signal=62% |
| 141 | GANDAL\_DEPRESSION\_DN\_PMID29439242 |  | 21 | -0.28 | -1.14 | 0.325 | 0.336 | 1.000 | 1195 | tags=48%, list=30%, signal=68% |
| 142 | GANDAL\_IBD\_DN\_PMID29439242 |  | 20 | -0.27 | -1.12 | 0.336 | 0.362 | 1.000 | 587 | tags=30%, list=15%, signal=35% |
| 143 | ALMSTEDT\_17Q\_MSIGDB |  | 123 | -0.17 | -1.10 | 0.348 | 0.386 | 1.000 | 1280 | tags=38%, list=33%, signal=55% |
| 144 | NB\_KI\_CLUSTER20 |  | 25 | -0.25 | -1.08 | 0.366 | 0.402 | 1.000 | 2387 | tags=80%, list=61%, signal=202% |
| 145 | WANG\_CLASSICAL\_PMID\_28697342 |  | 16 | -0.29 | -1.07 | 0.374 | 0.414 | 1.000 | 1052 | tags=44%, list=27%, signal=59% |
| 146 | DOROSZKO\_U3054MG\_CLUSTER\_8 |  | 153 | -0.16 | -1.06 | 0.420 | 0.431 | 1.000 | 1375 | tags=39%, list=35%, signal=58% |
| 147 | SUVA\_G2M\_PMID\_31327527 |  | 16 | -0.28 | -1.03 | 0.414 | 0.468 | 1.000 | 1581 | tags=56%, list=40%, signal=94% |
| 148 | RICHARDS\_NEFTEL\_CELL\_2019\_G2\_M |  | 16 | -0.28 | -1.03 | 0.436 | 0.465 | 1.000 | 1581 | tags=56%, list=40%, signal=94% |
| 149 | NB\_KI\_CLUSTER24 |  | 117 | -0.16 | -1.00 | 0.482 | 0.508 | 1.000 | 2890 | tags=86%, list=74%, signal=316% |
| 150 | RICHARDS\_VERHAAK\_CANCERCELL\_2010\_NEURAL |  | 22 | -0.23 | -0.97 | 0.502 | 0.549 | 1.000 | 296 | tags=18%, list=8%, signal=20% |
| 151 | NB\_KI\_CLUSTER7 |  | 158 | -0.14 | -0.97 | 0.540 | 0.551 | 1.000 | 3141 | tags=92%, list=80%, signal=439% |
| 152 | RICHARDS\_NOWAKOWSKI\_SCIENCE\_2017\_MGE\_IPC1\_UPREG |  | 214 | -0.14 | -0.96 | 0.562 | 0.552 | 1.000 | 393 | tags=14%, list=10%, signal=14% |
| 153 | FELDMAN\_2020\_S |  | 52 | -0.18 | -0.96 | 0.539 | 0.561 | 1.000 | 191 | tags=10%, list=5%, signal=10% |
| 154 | NB\_KI\_CLUSTER5 |  | 26 | -0.21 | -0.95 | 0.526 | 0.560 | 1.000 | 1242 | tags=38%, list=32%, signal=56% |
| 155 | DOROSZKO\_U3054MG\_CLUSTER\_4 |  | 144 | -0.14 | -0.91 | 0.628 | 0.611 | 1.000 | 1646 | tags=44%, list=42%, signal=74% |
| 156 | SUVA\_MES2\_PMID\_31327527 |  | 15 | -0.24 | -0.90 | 0.569 | 0.633 | 1.000 | 738 | tags=27%, list=19%, signal=33% |
| 157 | PARADA\_T2\_DN\_26461091 |  | 28 | -0.20 | -0.90 | 0.577 | 0.629 | 1.000 | 2298 | tags=71%, list=58%, signal=171% |
| 158 | RICHARDS\_NEFTEL\_CELL\_2019\_MES2 |  | 15 | -0.24 | -0.89 | 0.605 | 0.635 | 1.000 | 738 | tags=27%, list=19%, signal=33% |
| 159 | TF\_CENSUS\_TAIPALE |  | 427 | -0.12 | -0.87 | 0.744 | 0.651 | 1.000 | 937 | tags=24%, list=24%, signal=29% |
| 160 | NB\_KI\_CLUSTER22 |  | 63 | -0.13 | -0.76 | 0.769 | 0.783 | 1.000 | 1277 | tags=35%, list=32%, signal=51% |
| 161 | GAROFANO\_IAVARONE\_PPR\_SUBTYPE |  | 145 | -0.11 | -0.74 | 0.852 | 0.798 | 1.000 | 2213 | tags=58%, list=56%, signal=128% |
| 162 | NB\_KI\_CLUSTER13 |  | 61 | -0.13 | -0.70 | 0.803 | 0.823 | 1.000 | 2454 | tags=67%, list=62%, signal=176% |
| 163 | GAROFANO\_IAVARONE\_MTC\_SUBTYPE |  | 92 | -0.10 | -0.61 | 0.900 | 0.890 | 1.000 | 1594 | tags=40%, list=41%, signal=66% |
| 164 | NB\_KI\_CLUSTER12 |  | 25 | -0.14 | -0.61 | 0.914 | 0.886 | 1.000 | 2553 | tags=72%, list=65%, signal=204% |
Table: Gene sets enriched in phenotype **na **[plain text format]****
